# Material Binding Peptides for Nanotechnology 

**DOI:** 10.3390/molecules16021426

**Published:** 2011-02-09

**Authors:** Urartu Ozgur Safak Seker, Hilmi Volkan Demir

**Affiliations:** 1 Luminous! Center of Excellence for Semiconductor Lighting and Displays, School of Electrical and Electronic Engineering, Microelectronics Division; School of Physical and Mathematical Sciences, Physics and Applied Physics Division, Nanyang Technological University, 639798, Singapore; 2 Department of Electrical and Electronics Engineering, Department of Physics and UNAM, Institute of Material Science and Nanotechnology, Bilkent University, 06800 Ankara, Turkey

**Keywords:** material binding peptide, filamentous phage, nanotechnology, nanoparticles, self assembly

## Abstract

Remarkable progress has been made to date in the discovery of material binding peptides and their utilization in nanotechnology, which has brought new challenges and opportunities. Nowadays phage display is a versatile tool, important for the selection of ligands for proteins and peptides. This combinatorial approach has also been adapted over the past decade to select material-specific peptides. Screening and selection of such phage displayed material binding peptides has attracted great interest, in particular because of their use in nanotechnology. Phage display selected peptides are either synthesized independently or expressed on phage coat protein. Selected phage particles are subsequently utilized in the synthesis of nanoparticles, in the assembly of nanostructures on inorganic surfaces, and oriented protein immobilization as fusion partners of proteins. In this paper, we present an overview on the research conducted on this area. In this review we not only focus on the selection process, but also on molecular binding characterization and utilization of peptides as molecular linkers, molecular assemblers and material synthesizers.

## 1. Introduction

Phage display (PD) has been utilized as a powerful tool for the selection of ligands for many biological molecules [1,2,3,4]. In the case of peptides, the power of the phage displayed peptide libraries arose from the diversity of peptide sequences displayed on the phage coat protein, which have been demonstrated in numerous studies [5,6,7]. Additionally, by integrating the diversity enabled by PD peptide libraries, the evolutionary processes of biomolecules in biological systems can be mimicked through a forced laboratory evolution [8,9,10]. PD peptide ligands were selected and screened for proteins, small molecules, and peptides using a man-made evolutionary process. 

In addition to the soft materials found in biological systems, such as proteins, lipids and nucleic acids, hard tissues have also been discovered and systematically investigated for medical and technological applications [11,12,13]. Biological hard tissues were formed in diverse arrays of functionality and strength under extended durations of evolutionary stresses [14,15,16]. In the past few decades more attention was paid to formation mechanisms of biological hard tissues such as teeth [17,18,19], sea shells [20,21], bones and cartilages [22,23,24]. These studies emphasized the crucial role proteins play in the formation of biological hard tissues [25]. The synthesis of these biomaterials is uniquely controlled by specific biomolecules through molecular recognition and self-assembly [26,27].

The structure activity relationship of the biomaterial-forming proteins in living organisms has attracted increasing interest. In this context, some special proteins were extracted from organisms and shown to control the formation of biomaterials under ambient conditions, which revolutionized the area of biomaterials research. Hard tissue forming proteins and protein cascades are capable of forming materials in an unusual way compared to currently available synthetic methods. Naturally occurring proteins from various organisms were screened and extracted to observe *in vitro* their biomineralization and structural properties *in vitro*. Lustrin protein from *Haliotis rufescens* responsible for calcium carbonate mineralization [28], magnetite forming protein from magnetotactic bacteria localized in magnetosome of the bacteria [29], asprich protein from *Atrina rigida* [30], and silaffin protein isolated from *Cylindrotheca fusiformis* responsible for forming silica [31], are among the well known biomineral forming proteins. 

**Figure 1:**
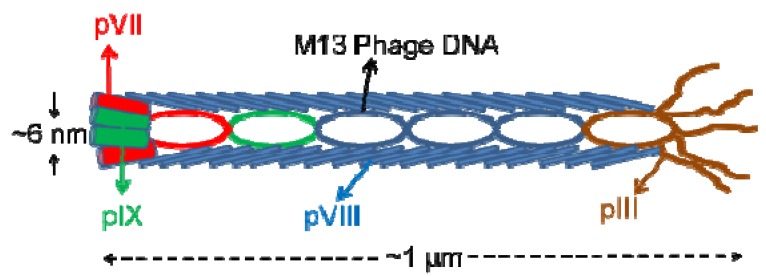
M13 phage with the coat proteins represented (note that the image is not drawn in scale).

Biologically available proteins and peptides were formed through evolutionary pathways and these proteins operate based a molecular recognition. To mimic the naturally occurring biomineral forming proteins and create artificial biomolecules for technological applications combinatorial biology techniques, namely the phage display and cell surface display technologies, were employed. First attempts for the selection of the inorganic material binding peptides were successfully made using the cell surface display by Brown *et al.* [32,33]. However, due to the limitations in the cell surface display, for the selection of material binding peptides, phage display has become the dominant combinatorial method. The advantage of the phage display peptide libraries is that phages can be genetically modified and phage clones can be utilized as molecular building blocks. Compared to bacterial cells and flagella, phages are more resistant to shear stresses, which may emerge during the binding of cells or phages on substrate material [34]. From a material science point of view, each of the phage clones displaying a different peptide motif is a different nanowire with different surface chemistry. For example M13 filamentous phage (Figure 1) can be considered a nanowire which is 1 µm in length and 6 nm in diameter [35]. Besides M13 phage display library, T4 and λ phage display libraries are also available; however, they are not used in the selection of materials binding peptides [36,37]. 

**Figure 2:**
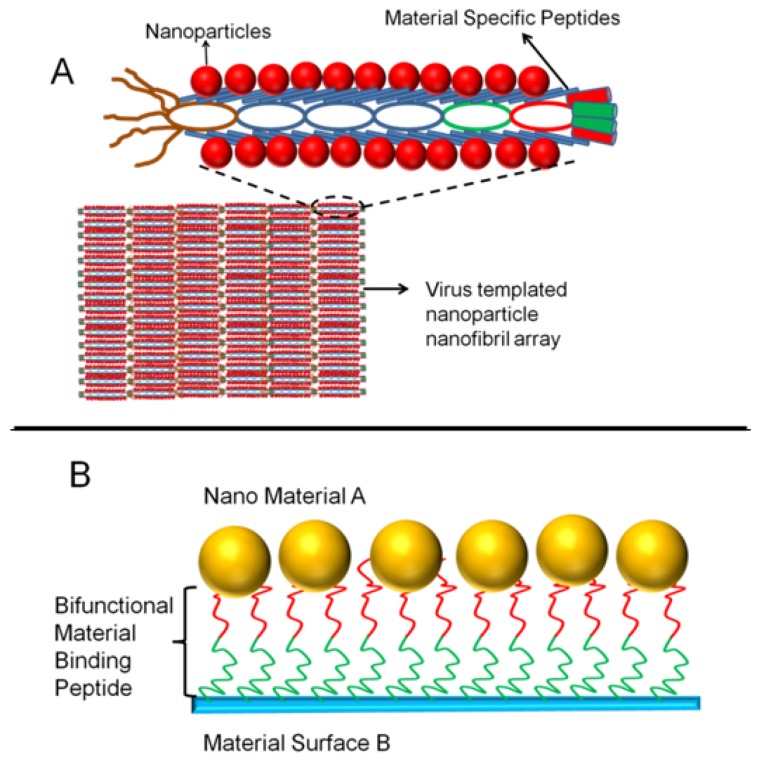
Two different approaches for the utilization of PD selected material binding peptides: (A) PD selected material binding peptides expressed on pVIII major coat protein used to assemble nanoparticles, (B) individually synthesized material binding peptides (in this case with dual functionality) used for the assembly and ordering of nanoparticles on a different material surface.

Phage displayed peptide libraries have been utilized for the selection of material binding peptides for a high number of materials. In the last decade peptides were selected for metals, metal oxides, metal compounds, polymeric materials, carbon materials, and semiconductors [38]. Following the selection of peptides, molecular characterization of these peptides has become an important tool for the robust and controlled design of peptide based material systems. In this manner, after screening the peptides, the material binding phages were purified and amplified. Later, using qualitative methods including fluorescence microscopy and colony counting, the binding affinity of phage clones was determined. Although these available methods have been useful for a quick classification of the phage clones, direct quantitative methods have been employed as well [39]. Once the selection and characterization are completed, PD selected peptides have been utilized for practical applications (Figure 2). To utilize the selected peptides in material systems, one possible approach is to basically use the whole phage body as the material binding agent [40], while the other is to synthesize the selected phage displayed peptides independently using solid state peptide synthesis method [41]. Yet another possibility is to use the selected inorganic peptides as fusion partners, to immobilize certain protein and enzymes on materials surfaces in an oriented and controlled fashion [42].

The selection of materials binding peptides from phage displayed peptide libraries has become a popular instrument, widely used for identification of novel peptidic linker molecules, which have been proposed and demonstrated for used in nanotechnological applications. This paper gives an overview of PD based biomimetic materials research, not only for the selection and characterization, but also for the utilization of these PD selected peptides as molecular linkers, materials synthesizers, and molecular assemblers into technological applications. 

## 2. Phage Display Selection and Screening of Material Binding Peptides

The screening and selection procedure for a peptidic ligand from a phage display library toward proteins, peptides, and other biological and chemical molecules has been well established since the early times of phage display [43]. However, it is essential to adapt combinatorial approaches to meet the needs of materials science. The design of binding experiments for the phage libraries is the key to the correct screening of binding phage clones. Preparation of the target material substrates must be done carefully. Substrate materials need to be characterized with surface analysis tools including X-ray photon emission spectroscopy, X-ray diffraction spectroscopy, scanning electron microscopy and transmission electron microscopy. Surface properties of a given material before PD must preferably be characterized for binding experiments. Additionally, inorganic materials can be found in many different forms; especially the chemical surface properties and crystal structure of the target materials play an important role during the binding process of phage clones. 

Different from the conventional phage display procedures, for the screening of the material binding phage clones, the phages libraries are directly brought in contact with the targets and they are not co-immobilized on a support. Target materials may be prepared in varying physical forms, e.g., in powder form, crushed sheets, single crystal or polycrystalline films. In addition, the buffer solution in which the substrate will be soaked needs to be optimized. The buffer solution must be inert to conserve the chemical surface properties of the material of interest. This requires a series of surface characterization right after treating the target material in chemically differentiated buffer solutions. The optimization of the buffer material is not only important for preventing corrosion and eroding of the material but also vital for avoiding the effects of non-specific binding of the phage clones. As a matter of fact, the surface hydrophobicity of the material of interest needs to be prevented by decreasing the surface tension at the solution-material interface through using an emulsifier (which is possible by adding a certain amount of detergents in buffer solution.) In most of the previous studies Tween has been used at a ratio of 1% (v/v) in the buffer solution during screening, this will allow for the suppression of non-specific interactions. [44].

**Figure 3:**
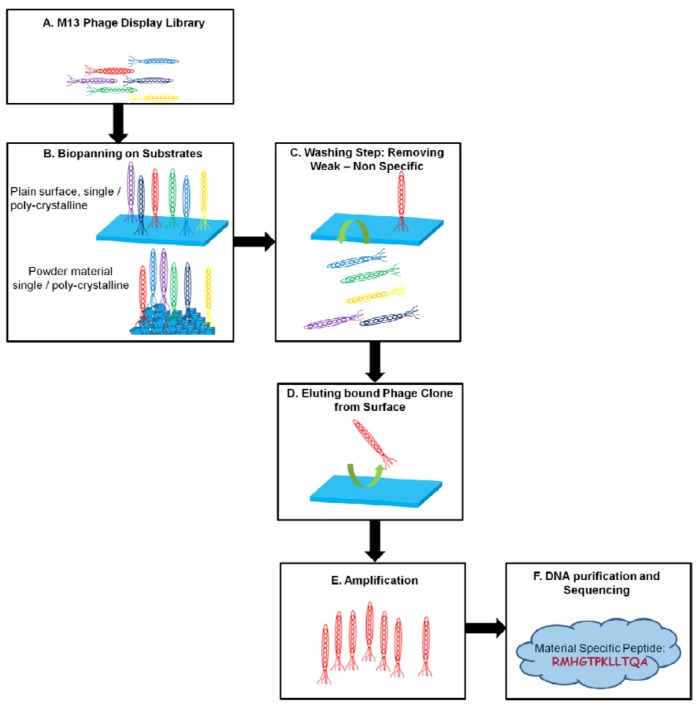
Schematic representation of PD selection of inorganic binding materials.

Depending on the type of the target material, there is a need for the optimization of incubation conditions of the phage clones. If the material is in powder form, then the peptide clones must be mixed (e.g., in a rotator) in a controlled way, but if the substrate is in sheet form, the phage clones can be incubated directly on the material surface for a certain time. Following the incubation of phage clones, to remove the weakly bound and non-bound clones, harsh washing needs to be carried out by modulating pH and ionic strength of the elution buffers. This step is especially critical to obtain strong binding clones. For this purpose the elution buffers must be carefully designed to harvest the strongest binder from the substrate surface. Following the phenotype based selection of phage clones, the genotypes of the phage clones were determined. The schematic presented in Figure 3 gives an overview of the phage display selection of material binding peptides.

In most of the previous studies towards discovery of material specific peptides, commercially available M13 phage display libraries were used [45,46,47]. A schematic of a M13 phage is represented in Figure 1. In M13 phage libraries the pIII phage minor coat protein is carrying the insert coding the peptide. The insert was displayed as constrained heptamer or dodecamer linear peptides. The 7-mer peptides were expressed in constrained loop formed through a disulfide bridge. The linear peptides were fused to the pIII protein through a linker sequence which is –SGGG in the linear phage libraries and –SGGGC-XXXXXXX-AC in the case of 7-mer constrained peptides. 

## 3. Selection and Characterization and Potential Uses of the Material Binding Peptides

The PD selected materials binding peptides can be classified as metal, metal oxide, metal alloy, metal compounds, mineral, semiconductor, carbon material and polymer binders. In this part, the phage display selected material binding peptides from literature are listed in corresponding groups. To explore the utilization of these selected peptide sequences, a deep understating of both structural features and binding affinity has developed, so here we have also included the studies towards characterization of peptides. The efforts for the characterization of these peptides were carried out by using both quantitative and qualitative methods. Most of the affinity characterization studies were related to the structural features of the peptides, like in protein-protein interactions, structure-activity based research results were demonstrated. 

### 3.1. Metal, Metal Oxide, Metal Alloys and Metal Compounds Binding Peptides

Metals and metal compounds have been utilized in many technological applications including biomedical ones [48,49]. Most of the metal surfaces are well defined by means of chemistry and morphology. The first example of the material binding peptide from a combinatorial peptide library is iron oxide binding peptides selected from a cell surface display library [33] and later gold binding peptides were isolated from a bacterial cell surface display library by Brown, whose studies pioneered the isolation and utilization of solid binding peptides [32]. The gold binding peptide screened and selected by Brown, was later utilized in human studies, and became a well characterized peptidic linker for immobilization of nanomaterials [50], materials synthesizer [51], and protein fusion partners for oriented protein immobilization [52]. 

A phage display selected gold binding peptide, which is positively charged and selected using gold powder as the target material, was first reported by Whaley *et al*. [40]. This peptide was expressed on the phage coat and then gold nanoparticles were assembled on the phage via the selected peptide. Similarly, another gold binding peptide was isolated by the same group; this time the selection was carried out on a thin gold film. The peptide was displayed on the pVIII phage major coat protein and used to assemble gold nanoparticles while additionally expressed peptides on the same coat protein were used to co-assemble with CdSe nanocrystals on to create 2D optical assemblies [53]. Both peptides were characterized using plaque counting on solid media for their binding affinity on gold. Naik *et al*. reported another gold binding peptide, however this was not specially selected towards gold surfaces. AG3 peptide was originally selected for silver surfaces but utilized also for gold nanoparticle synthesis [54,55]. Kim *et al.*, has recently isolated gold binding peptides with distinct nanoparticle formation capabilities, using PD approach and gold powder as the target material [56]. They picked one of the peptides, called Midas-2, which they utilized for the gold nanoparticle formation. Their first set of nanoparticle synthesis yielded poly-disperse gold nanoparticles. While probing the relationship between the primary structure of the binding peptide and shape-size of the synthesized nanoparticles, they carried out a point mutation based analysis by replacing each amino acid position with glycine. The Midas-2 mutant, Midas-11 was shown to form large gold platelets as wide as 24 µm with a thickness of 30-150 nm. These gold nano-platelets were formed in hexagonal, trigonal shapes [56]. This work by Kim *et al.* is a good example for controlling the size and shape of nano-structures by tuning the structural properties of example specific solid binding peptides; a similar type of conclusion was also previously presented in the work by Brown *et al*. [32,51]. 

Like gold, silver is another frequently studied material. Silver binding peptides were screened and selected toward acid etched nanosized silver particles. The selected silver binding peptide ligands, AG3 and AG4, which are both 12-mer peptides, were determined as the predominant silver binding peptides upon their capability to form silver nanoparticles with a strong localized surface plasmon resonance (LSPR) band.

**Figure 4:**
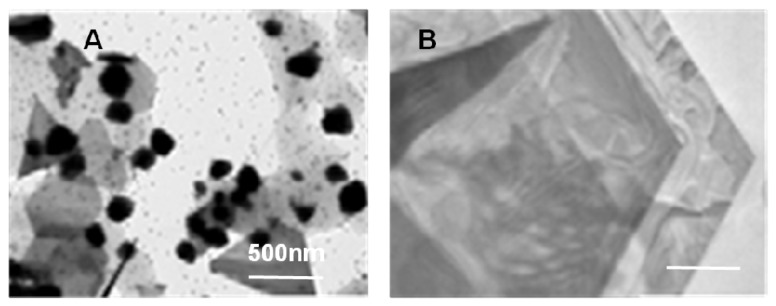
Gold nanoparticle formation in the presence of Midas-2 peptide, and gold platelet formed in the presence of Midas-11 peptides. Reprinted from [56] with permission from Elsevier. Copyright (2010).

AG4 was also immobilized in a microfluidic system made of an elastomer to demonstrate its ability to grow arrays of silver nanoparticles [55]. The solution structure of the silver binding peptide was investigated as well; this was achieved using nuclear magnetic resonance (NMR). The data revealed that, during the synthesis of silver nanoparticles, multiple AG4 peptides are stuck on a nanoparticle and, Leu5, Phe6, and Arg7 residues were suggested to be the contact points for binding [57]. 

The exploration of silver binders was further continued by proposing a new polymerase chain reaction (PCR)-based panning for phage displayed peptides, which resulted in many new silver binders. These binders were characterized for their interaction with the sliver nanoparticles, using an agarose gel based separation of the particle-peptide complex [58]. Moreover, AG4 was shown to control the orientation of maltose binding protein (MBP) on silver nanoparticles, when it was genetically fused to MBP, which was also validated in a surface enhanced raman spectroscopy (SERS) study. Also, an order in magnitude enhancement of the affinity of MBP AG4 fusion towards silver surface was achieved compared to MBP native [59]. 

Platinum and palladium binding peptides were first reported by Sarikaya *et al*. [60], both isolated from 7-mer constrained phage display library. Molecular modeling studies of the platinum binding peptides indicated that a core domain may be responsible for the binding of the peptides, which is –TST- region in strong binding platinum binding peptides [61,62]. The effect of elongation in the peptide sequence constrained conformation on the adsorption of the peptides onto platinum surface. The results indicated the importance of the orientation of the interaction points on the peptides, which is directly related to the conformational control of peptide affinity [63,64]. A proof of concept application of the platinum binding peptides was demonstrated to immobilize a photoresponsive fluorescent probe on a platinum surface for sensing purposes [65]. Moreover, the possibility of using platinum binding peptides was successfully shown to create biologically active surfaces for enhanced biocompatibility for biomedical applications [66].

**Figure 5:**
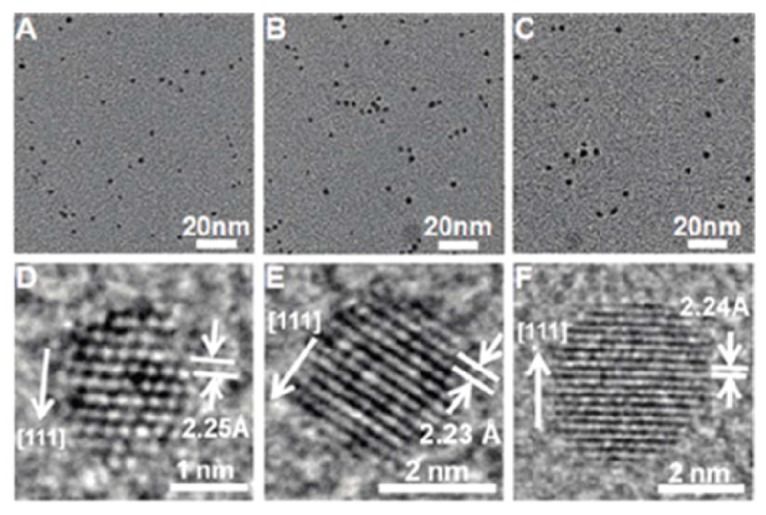
Ultra small platinum nanoparticles formed in the presence of platinum specific peptides, each image is taken at different time during nanoparticle formation, A (10s), B (60s) and C (5h). Reprinted with permission from [67]. Copyright 2009 American Chemical Society.

Li *et al*. have also screened and selected platinum binding peptides from 7-mer phage display libraries using micrometer size platinum particles as the target substrate. After successful biopanning, the strong platinum binder P7A was used to synthesize ultra small platinum nanoparticles, which have a narrow size distribution between 1.5–3.5 nm, which is presented in Figure 5 [67].

Another noble metal, palladium, is also used as a catalyst in chemical reactions; in particular palladium nanostructures were found promising as catalysts for fuel cells or environmental applications [68]. To initiate a new route for the green synthesis of palladium nanoparticles, peptide ligands were screened and selected from a 12-mer PD library. Like the platinum nanoparticles, synthesis of ultra small palladium nanoparticles, with a size of ~2 nm, was achieved by employing Pd2 and Pd4 peptides. More recently, Chiu *et al*. have also demonstrated palladium nanoparticle formation utilizing a phage display selected peptide, from a 7-mer PD library [69]. 

Nian *et al.* have also carried out a novel and challenging bio-panning method for the selection of Pb^+ ^binding peptides. In this study iminodiacetic acid (IDA) adsorbed bead columns loaded with Pb^+ ^and other metal ions were used to isolate Pb^+ ^binding phage clones, while avoiding cross-binding phage clones with unwanted metal ions from a cyclic septameric PD library. The library was first cleaned from the non-specifically IDA binding clones. The eluted binders were subjected to another biopanning using Cu^2+^, Ni^2+^, Co^2+^ , and Fe^3+^ immobilized IDA beads. This enables picking of phage clones which are only specific to Pb^+^, but not the other metal ions mentioned above [70]. 

Not precious as gold, silver or platinum, steel is another widely used material in many industries and the most important problem is that it is open to corrosion and can be eroded easily. With the aim of suggesting a new method to prevent steel and alumina from corrosion, Zuo *et al.* reported mild steel and aluminum binding peptides. The most notable part of their study is their way of biopanning during the selection. They used a special corrosive solution to elute weakly bound peptides. This approach can be extended to future studies for selecting peptides with specific aims [71]. 

Titanium is a widely used biomaterial because of its biocompatibility. However, as a popular implant material, surface functionalization of titanium is challenging. To solve existing problems of titanium surface functionalization, peptides were screened from 12-mer and 7-mer PD libraries. Liu *et al.* selected peptides directly for a commercially used material cp-Ti. Using confocal microscopy they observed surface bound clones, and characterized the binding of phages on cp-Ti surface [72]. Moreover, Meyer *et al.* carried out a more complete study following the selection of 12-mer titanium binding peptides. They synthesized integrin binding domain functionalized RGD derivatives of their titanium binders and successfully demonstrated how the endothelial cells preferably grow on titanium binder-RGD decorated titanium surface [73].

As common metal oxides silica and titania are used in numerous applications ranging from optoelectronic devices to biomedical systems. Consequently, many groups have focused on these materials to screen and select silica and titania binding peptides. Naik *et al.* reported the first silica binding peptides isolated from a phage display library with their unique capability to precipitate silica from silicilic acid, which are rich in arginine or histidine amino acids [74]. Different from Naik *et al.* another group of silica binding peptides were screened for their affinity towards a single crystalline quartz surface, which are rich in proline amino acids [75]. A quantitative analysis of the strongest binders among the single crystal quartz binding peptides was carried out using surface plasmon resonance spectroscopy (SPR). The peptides QBP1 and QBP2 were found to have equilibrium binding constants of 0.12 × 10^6^ and 1.2 × 10^6^, respectively. The effect of making concatamers for a increased affinity was also tested for these peptides. However, this was confirmed only for QBP1, but not for QBP2. This type of behavior was considered as a sign for a complex binding mechanism of the solid binding peptides. Here primary structure and conformational transition of the peptide upon adsorption at solid-liquid interface are to play a crucial role, contributing complexity of the process [46]. 

Interestingly, silica binding peptides were screened using different strategies for different types of silica targets. For example, in a study by Etheshola *et al.*, silica binding peptides were screened against a thermally grown silica substrate, a technologically common substrate which is a smart choice for peptide selection considering that most of the silica surfaces on devices and sensors are actually thermally grown. The affinity of the silica binding peptides to thermally grown silica was classified according their biopanning yield, which provides an overall qualitative idea for degree of binding. Similar to those peptides selected for single crystalline quartz surface, thermally grown silica binding peptides are also rich in proline residues [76]. As another type of silica substrate, Chen *et al.* screened silica binding peptides towards silica nanoparticles. The resulting peptides, which are rich in charged amino acids are conformationally restricted [77].

In a complementary study, by employing point mutations a diverse conformational space of peptides was investigated, and the importance of the peptide conformation on the binding affinity was demonstrated [78]. Chen *et al.* also aimed to screen titania binding peptides in the same study, but surprisingly they obtained with a sequence that showed a high affinity both towards titania and silica surfaces. This kind of dual affinity behavior was previously observed in another study conducted by Shiba *et al.* on TBP1 peptide [41,79]. This observation may also be expected due to the close chemical characteristics of both metal oxide surfaces [80]. TBP1 peptide was selected from a 12-mer PD library using atomized titania as the substrate. The affinity dissociation constants of TBP1 peptide for silica and titania were found to be close (13.2 µM and 11.1 µM respectively), which shows that this peptide is not selective between these two metal-oxide surfaces. If the cross-specificity experiment, the phage particles harboring TBP1 was also found to be nonselective between titania and silica.

TBP1 peptide has been also exploited in proof-of-concept studies on nanomaterial assembly. TBP1 was fused to the N-terminus of apoferritin. Ferritin-TBP1 fusion was tested for its binding affinity and biomineralization activity. The affinity constant for this fusion toward titania was calculated to be 3.82 nM [81]. Nanocrystal filled ferritin-TBP1 was utilized as a molecular building block to create layer by layer assembled nanostructures. TBP1 facilitated the biomineralization of titania and silica interlayer between assembled nanocrystal filled ferritin-TBP1, so that a multilayered structure was constructed [82]. Similarly, the selective formation multilayer of nanocrystal filled ferritin on titania strips but not on platinum was succeeded, using a platinum patterned titania film [83].

**Figure 6:**
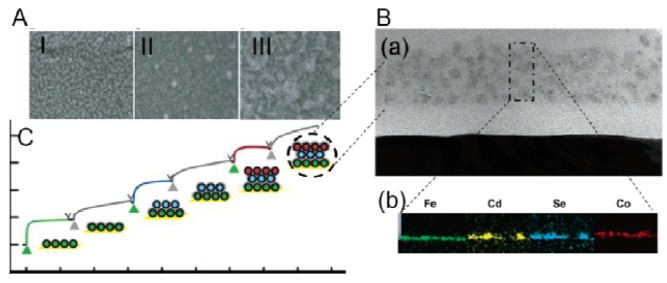
Layer by layer (LbL) assembly of Fe, Cd, Se and CO filled ferritin-TBP1 molecules: (A) SEM images of titania surface (I), after decoration with Ferritin-TBP1 (II), and after biomineralization of silica interlayer (III). (B) intersection of the constructed LbL (a), EDS mapping of metal filled ferritin-TBP1 after LbL assembly (b), (C) The QCM-D signal of the construction of ferritin-TBP1 and silica interlayers. Adapted with permission from [82]. Copyright 2009 American Chemical Society.

Dickerson *et al.* reported titania binders, which were selected for rutile type single crystaline (100), (101) and (111) titania surfaces, and they were also shown to be capable of forming titania nanoparticles with anatase type monoclinic crystal domains. In this study, the importance of charged residues in binding process of the peptides was also discusses [84].

In addition to experimental studies oxide binders were investigated in comprehensive computational studies. As an example, the importance of different effects other than electrostatic interactions such as pi-pi interactions and hydrophilic interactions were shown in silica binding peptides, which provided a good source of information towards understanding possible interactions during the binding process [85,86]. 

As discussed above, most of the previous studies found that some of the titania binders also adhere on silica surfaces. A remarkable study opened a new avenue to eliminate the possibility of dual affinity of titania binders. They proposed a subtractive biopanning approach, similar to the study of Nian *et al.*, where the silica binders were removed from the PD library by incubating them with silica nanoparticles. Therefore, they eliminated the possibility for a silica binder to be among the remaining of the 12-mer phage clones. The final titania binders were demonstrated to precipitate titania from potassium bis(oxalato)oxotitanate(IV) but not silica from silicilic acid [87]. 

Zinc oxide is used in many optical and optoelectronic applications was a wide band gap material [88]. Umetsu *et al.* made the selection of ZnO binding peptides from a 12-mer phage displayed library using micrometer sized ZnO particles as the target substrate, and further showed that ZnO binding peptides can discriminate ZnO from ZnS. By immobilizing ZnO binding peptides on a gold plate, the formation of ZnO nanoparticles in a flower-like shape was tuned. In a similar study, same effect of the peptides on the formation of ZnO nanoparticles with distinct shapes and sizes on a biopolymer surface was explored [89,90]. These studies proposed a novel way to create fluorescent ZnO particles with unique morphologies compared to available low temperature synthesis approaches, which needs a series of complex chemical reactions [91]. Veruls *et al.* also selected 12-mer ZnO binding peptides, which were used as a fluorescent probe to examine the quality of the ZnO coatings applied on galvanized steel. According to this study specific fluorescent labeled peptides were shown to adhere only into cracks of ZnO, and using a simple fluorescent microcopy the surface quality check was proposed [92]. 

Besides silicon oxide, titanium oxide and zinc oxide, the phage display approach was also utilized to discover material binding peptides for other metal oxides; however, the number of these reports for the rest of oxide materials is smaller. These possible reasons for a lower number of such studies are that these metal oxides are not as widely used and/or that peptides are not suitable for surface functionalization under their processing. Iridium oxide is one of the oxide materials; for which a binding peptide from a 8-mer PD library was selected. In this study iridium oxide nanoparticles were assembled on the phage body surfaces, where iridium oxide binding peptides were on coat protein pVIII. As a co-assembly, porphyrin molecules were also chemically grafted on the coat protein of M13 phage as a photosensitizer. These were utilized as a photocatalytic system for light driven water oxidation [93].

**Figure 7:**
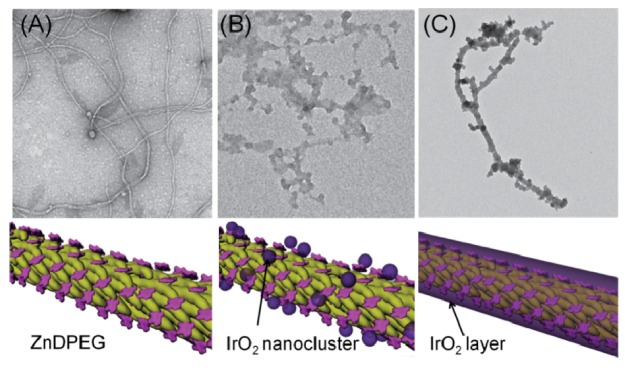
Construction of phage particles decorated with iridiumoxide nanoparticles co-assembled with ZnDPEG. (A) ZnDPEG grafted phage (B) iridium oxide nanoparticle nucleation via iridumoxide binding peptides on phage coat (C) final form of phage nanowire coated iridiumoxide. Reprinted by permission from Macmillan Publishers Ltd: Nature Nanotechnology [93], copyright 2010.

Germanium precipitating phage clones were isolated with the aim of forming biochemically controlled germanium nanostructures [94]. Similarly, 12-mer peptides for hematite surfaces were selected. Based on molecular modeling studies, a general oxide binding motif of –SPS- and –SGS- was proposed [95]. However, considering the variety of the metal oxide binding peptides, it may be difficult to define a single consensus sequence for all of the metal oxide binders. But it may be plausible to argue the presence of some amino acids harboring charged groups on their side groups may which may contribute to the affinity of the oxide binding peptides. 

Other than metals and metal oxides, some peptides were also screened to synthesize metal alloys and some special metal compounds that were utilized in actual applications. For this, peptide ligands were screened and selected for metal alloys composed of Fe-Pt. The metal alloy building activity of the selected phage clones were demonstrated. The synthesized alloys were found to exhibit a strong ferromagnetic characteristic, a similar type of approach was employed to create Co-Pt magnetic alloys using cobalt binding peptides as biotemplate [96,97]. 

Ferroelectric materials e.g., barium titanate and perovskite have been investigated for selection of PD based peptidic ligands. Ahmad *et al*. used the selected peptides for the synthesis of barium titanate nanoparticles [98]. Reiss *et al.* employed the perovskite binding peptides for surface functionalization of perovskite, and noted no significant chemical alteration of the perovskite following the surface functionalization [99]. 

### 3.2. Semiconductor Binding Peptides

In the past two decades incredible progress had been made in semiconductor nanocrystals synthesis. Today semiconductor nanocrystals find a wide range area of applications in areas from optics to biosensing [100,101]. PD libraries were used in screening and selection of semiconductor binding peptides. 

GaAs binding peptides were selected using a 12-mer PD library, and these first GaAs binding peptides were also demonstrated for their selectivity among silica, gold and GaAs materials. The GaAs binding peptide selection also initiated selection of PD based peptides for a number of other semiconductors. A detailed TEM analysis was also included for the exact demonstration of selectivity of these GaAs peptides [40]. Similar approaches were later used by Estephan *et al.* for the exploration of GaN specific peptides, for which they characterized the binding affinity of the peptide using a atomic force microscopy (AFM) molecular force measurement tested the cross specificity on a silica/GaN substrate through fluorescence microscopy of labeled peptidic aptamers [102]. 

For the selection of II–VI semiconductor binding peptides, ZnS and CdS single crystals were used for biopanning of 7-mer and 12-mer PD libraries. Phage particles for both of the materials were classified regarding their adhesion on single crystals. A7 peptide was determined as a strong binder for ZnS. Using the phage clone harboring the A7 peptide formation of nucleated nanocrystal structures of ZnS was also realized. The resulting ZnS crystals upon catalysis of A7 peptide was investigated in details using high resolution transmission electron microscopy (HRTEM) analysis. Diffraction patterns from TEM analysis revealed the regulation effect of A7 peptide on the crystal formation of ZnS. A7 phage clones were further utilized to assemble nanocrystals in film-like supramolecular structures by using phages as a network formed by nanofibrillar structures [103,104]. Estephan *et al.* screened a 12-mer phage display library for the exploration of CdSe, GaSb, GaAs, ZnTe, ZnSe, GaN, InAs, GaAs and InP binding phage clones, where they carried out their selection in six rounds and extracted phage clones from each different round of selection. Interestingly, a peptide named P1was discovered from different rounds of the selection as a putative binder for most of the above listed semiconductor surfaces. To eliminate the possibility that the P1 peptide cloning phage is overexpressed in the M13 phage library, they also conducted a detailed binding experiment using mass spectroscopy (MALDI-TOF), which depends on the mass analysis of the bound synthetic peptides extracted from the target surface. They also demonstrated the importance of the solvent used for the correct assembly of solid binding peptides to their targets [45,105]. Cross specificity and affinity of the semiconductor binding peptides was studied using AFM by Goede *et al.* to characterize the interaction a peptide adhesion coefficient was calculated. A strong relationship between the peptide structure and affinity was shown upon the change in the adhesion coefficient [106].

### 3.3. Mineral Binding Peptides

In Nature many peptides and proteins were evolved to function in the synthesis of bio-mineralized hard tissues [28,31,107]. Among these hard tissues, teeth and bones were well studied as mentioned above, and the main constituent of these tissues is hydroxyapetite (HA) mineral, which is commonly found in different crystal structures at the different parts of teeth and bones. Although some of the HA binding proteins were characterized in detail, they cannot be easily produced and fused with some other functional proteins, especially for surface functionalization or fluorescent probing. To utilize in biomedical applications, aptamers for HA were screened and selected from 12-mer and 7-mer PD libraries. 

Gungormus *et al.* selected and characterized HA binding peptides, namely HABP1 and HABP2, which are strong and weak binders, respectively. They tested the effect of HA binding peptides on the biomineralization of calcium phosphate. The results were promising, both of the binders can produce minerals as crystals, and however the weak binders can trigger smaller crystals in size [108]. Other HA binding peptides were screened and tested for their selectivity for different calcium phosphate minerals by Roy *et al*. HA binding peptides were observed to be selective between amorphous calcium phosphate and hydroxyapetite, and to exhibit high affinity towards human tooth surface which rich in hydroxyapetite minerals [109]. 

As another calcium compound, calcite was also investigated to control its formation using peptides. Calcite binding peptides were isolated from phage display libraries separately by Gaskin *et al*. and Li *et al*. Both sequences were found to control the calcite crystal formation. In both cases peptides deterred transformation from vaterite to calcite [110,111].

### 3.4. Carbon Materials Binding Peptides

Carbon materials have found a vast range of use in technological applications [112,113,114,115]. The first carbon material binding peptides were selected for carbon nanotubes (CNTs). CNT binding peptides opened a new avenue for the surface functionalization, sorting and dispersing of CNTs. This was an innovative step towards developing CNT based biohybrid applications [116]. Both in experimental and modeling studies, it was found that histidine and tryptophan rich residues are important in the interaction of CNT binding peptides with the CNT surfaces [116,117].

**Figure 8:**
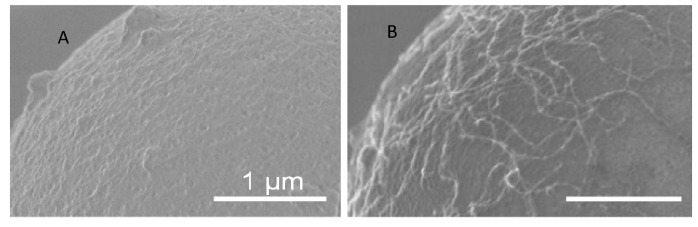
Decorating of the surface of a microsphere with CNT using CNT binding peptides. (A) Microsphere surface in the absence of CNT binding peptide and (B) adhesion of CNTs on CNT binding peptide decorated microsphere Reprinted by permission from Macmillan Publishers Ltd: Nature Materials [116], copyright 2003.

Single walled carbon nanohorns were also targeted by phage display to elute new peptidic ligands. These isolated ligand molecules were also classified and investigated from a structural point of view. The results suggest that of labile structural properties of the selected peptides play a crucial role in the binding of the peptides [118]. C_60_ recognizing peptides were also screened and selected using PD library, for which fluorescence microscopy analysis yielded string binders. C_60_ binding peptides promise a good strategy for surface functionalization of C_60_ [47].

### 3.5. Polymer Binding Peptides

Material binding peptides have been routinely screened and selected for solid inorganic materials. However, only recently PD libraries have been employed for polymers and polymer binding peptide ligands have successfully been screened for the functionalization of polymer surfaces. The first polymer binding peptides were isolated for chlorine-doped polypyrrole, which is a conductive polymer used in electronic and biomedical applications. Strong binder selected from 12-mer PD library was independently synthesized and, using atomic force spectroscopy, the binding affinity of T59 peptide and its variants was tested. The results suggested a strong binding with the polymer surface. T59 peptide was hybdrized with an integrin binding peptide GRGDS. Its binding and unbinding measurements revealed a strong binding within the peptide-polymer surface. Moreover the RGD integrated T59 was used to enhance cell proliferation on the polymer surface [119]. After the report this first polymer binding peptide, Serizawa *et al.* reported on a PD selected 7-mer peptide, which can recognize the stereoregularity on isotactic poly(methylmethacrylate) (it-PMMA) surface. In this work, during the binding analysis of phage clones, -RPTR- sequence was detected as a core motif in this peptide for the affinity. In a later study, the binding kinetics and affinity of the ti-PMMA binding peptide were examined. The equilibrium binding constants of this peptide was found to be *K_eq_* of 7.6 × 10^5^ M^−1^ [120,121], which is almost one order of magnitude higher compared to the peptide selected for poly-L-lactate [122]. 

Polystyrene (PS) is a widely used polymer in many areas of biomedical research applications. Aiming to create a new PS surface modifying agent, Serizawa *et al.* screened and selected peptides for syndiotactic polystyrene (sPS). The resulting phage clones can strongly adhere on PS surfaces. Similar to their previous studies, they also demonstrated that these peptides can recognize the stereoregularity of the polymer surfaces, and concluded that this property can lead to differentiation of nanostructural changes in the polymer films surface by using these peptides [123,124]. Cellulose binding peptides were also screened and selected as the only naturally occurring polymer binding ligand with high affinity, which may further allow cellulose fibers to be utilized in biomedical studies [125]. 

## 4. Examples of Material Binding Peptides Utilization toward Practical Applications

In decade, studies on material binding peptides have led to the formation of a large collection of PD selected material peptides, a list of which is presented in Table 1, along with available binding affinity constants and special applications.

**Table 1:** Strong Material Binding Peptides from Literature.

Material of Interest	Peptide Sequence	Notes
**Gold**	^#^VSGSSPDS [53], ^#^LKAHLPPSRLPS [130]	Gold nanoparticle (NP)assembly
	^*^TGTSVLIATPYV [56]	Gold NP synthesis
**Silver**	^*^AYSSGAPPMPPF [131]	Ag NP synthesis
	^*^IRPAIHIIPISH, ^*^WSWRSPTPHVVT [58]	Ag NP synthesis
**Silica**	^#^MSPHPHPRHHHT, ^#^RGRRRRLSCRLL [74]	Silica precipitation
	RLNPPSQMDPPF, QTWPPPLWFSTS [75]	SPR *K_eq_*(M^−1^): 0.12 × 10^6^, 1.24 × 10^6^
	HPPMNASHPHMH, HTKHSHTSPPPL [132]	
	CHKKPSKSC [77]	LacI fusion QCM-D *K_eq _*(M^−1^): 2.46 × 10^8^ [133]
**Titania/ Titanium**	^*^RKLPDAPGMHTW [79,81]	Depletion assay K_eq _(M^−1^): 7.58 × 10^4^
	^*^YPSAPPQWLTNT, ^*^STPLVTGTNNLM ^*^QSGSHVTGDLRL, ^*^ATTLHPPRTSLP[87]	Subtractive biopanning
	^#^SCSDCLKSVDFIPSSLASS [73]	ELISA K_eq_(M^−1^): 4 × 10^6^
	^#^LNAAVPFTMAGS [92].	
	^#^ATWVSPY [72]	Confocal microscopy
	^*^RKKRTKNPTHKLGGGW, ^*^KSLSRHDHIHHHGGGW^*^TQHLSHPRYATKGGGW [84]	
**Zinc Oxide**	^*^EAHVMHKVAPRP [89], ^*^GLHVMHLVAPPR [90]	ZnO NP synthesis
	^*^VRTRDDARTHRK [92]	Surface Quality Control
**Iridium Oxide**	^#^AGETQQAM [93]	NP formation,co assembly
**Iron Oxide**	^#^LSTVQTISPSNH [95]	
**Germania**	^*^TGHQSPGAYAAH, ^*^SLKMPHWPHLLP [94]	NP network formation
**Platinum**	^*^CPTSTGQAC, ^*^CTLHVSSYC	SPR *K_eq_*(M^−1^): 3.4 × 10^6^, 9.0 × 10^4^,
**Palladium**	^*^QQSWPIS [134], ^*^NFMSLPRLGHMH [69],	Pd NP synthesis
	^#^SVTQNKY, ^#^SPHPGPY, ^#^HAPTPML [5]	Phage ELISA
**Aluminium**	^#^VPSSGPQDTRTT, ^#^YSPDPRPWSSRY [71]	
**Stainless Steel**	^*^MTWDPSLASPRS [92]	Surface Quality Control
	^*^ATIHDAFYSAPE, ^*^NLNPNTASAMHV [71]	
**Fe-Pt Alloy**	^#^HNKHLPSTQPLA, SVSVGMKPSPRP, VISNHRESSRPL [96]	FePt NP synthesis
**Cobalt**	^#^HSVRWLLPGAHP, KLHSSPHTLPVQ, [58]	CoPt NP synthesis
**Hydroxyapatite**	^#^SVSVGMKPSPRP [109]	
	^*^CMLPHHGAC [108]	Mineral synthesis
**Polymers**		
Poly(L-lactide)	^*^QLMHDYR [122]	SPR K_eq _(M^−1^): 6.1 × 10^4^
Polypyrrole	^*^THRTSTLDYFVI [119]	AFM analysis
it-PMMA	^*^ELWRPTR [135]	SPR K_eq_ (M^−1^): 7.6 × 10^5^
sPS	^#^YLTMPTP	ELISA K*_eq_*(M^−1^): 2 × 10^11^
**Semiconductors**		
GaAs- InP	^#^AQNPSDNNTHTH [40], ^*^SVSVGMKPSPRP [105]	
ZnS- PbS- CdS	^#^CNNPMHQNC, ^#^QNPIHTH, ^#^CTYSRLHLC [103]	

^#^ On phage particles; ^*^ independently synthesized using FMOC solid peptide synthesis.

Thus far PD selected peptides have been utilized in nanotechnology and biotechnology applications. Because the area has been emerging just about for a decade now, and there is a still a large room for applications. To date, materials binding peptides have been utilized as molecular assemblers, material synthesizers, and genetic fusion partners of proteins and enzymes. Some of these uses and potential applications are summarized above.

Nanoparticle synthesis is an area of application in which PD selected peptides are widely used. These peptides are capable of synthesizing nanoparticles made of the materials they are selected. These peptides are presented above. During the nanoparticle synthesis, peptides alsoserve as catalysts for the particle formation in the case of metal and metal compound based nanoparticles. However, for the formation of minerals, material binding peptides act as regulating agents that to restrict the growth of the mineral crystal. In this case, materials binding peptides are capable of controlling the morphology of the synthesized nanomaterials and micromaterials. This is a desire property by the material scientists for the invention of novel material synthesis routes. To date material binding peptides have been used in the nanoparticle synthesis of Au [51,54,56], Ag [55,126], Pt [5,67], Pd [69], Fe-Pt and Co-Pt metal alloys [58,96], SiO_2_ [46,74,76], TiO_2_ [77,79,84,87], barium titanite [98], zinc oxide [89,127], and germenia [94] as well as minerals of calcite [110,111], mica [128] and hydroxyapetite [108]. In addition, several case studies for the genetic fusion of some PD selected material binding peptides are described above [59,81,129]. 

PD selected material specific peptides have been widely used as the molecular linker molecules, where they have been commonly utilized to assemble nanomaterials. For this purpose, both independently synthesized peptide linkers and phages were used. In the case of using phage clones, the peptide is expressed generally on the pVIII major coat protein because of the high copy number of this protein on the phage body. Kacar *et al.* used a silica binding peptide, QBP1, derived from PD selected silica binders using computational tools, to assemble quantum dot nanocrystals and flourescein in the shape of arrays, which after potential use as optically active layers [136]. 

Similarly, the same group demonstrated gold binding peptide as a linker to control the distance between quantum dots and nanofabricated gold nanoarrays to enhance the fluorescence via near-field plasmonic coupling [137]. Nochomovitz *et al.* also built a bifunctional peptide that consists of a gold binding peptide and carbon nanotube binding peptide connected via a linker. This bifunctional peptide was used to functionalize the silica surface either with carbon nanotubes or with gold nanoparticles, and similarly the gold surface was functionalized using silica nanoparticles [50]. In a recent study, Cui *et al*. successfully demonstrated coupling of the graphene surface with gold nanoparticles using such a bifunctional linker molecule [138]. Similarly Kuang *et al.* functionalized the single walled carbon-nanotube surface with a SWNT binding peptide, which is coupled with a TNT binding domain (honeybee odor binding protein) [139]. Both platforms were demonstrated as a candidate for a TNT sensor, which relies on preparation of a SWNT field effect transistor. Other groups used filamentous phage clones, expressing selected material binding peptides as a nanowire platform to assemble nanomaterials to create ordered assemblies. Ki Tae Nam *et al.* demonstrated utilization of a phage clone to express a specific gold binding peptide and a non-specific cobalt nucleating motif to create Co-Au hybrid nanowire [130]. The same approach was previously shown to be effectively used in the synthesis of single crystal ZnS and CdSe nanowires as well as free standing ordered FePt and CoPt nanowires [140]. Recently, putting all these together, a notable challenge was achieved by means of which multiple virus genes encoding different material binding peptides was utilized in the formation of an actively operating lithium-ion battery [141]. 

## 5. Conclusions

To date PD libraries have been successfully applied for the selection and screening of material binding peptides grouped as follows: those for metals, metal compounds, metal alloys, semiconductors, minerals, and polymers. Today PD is a well established tool for the selection of ligand molecules for biological molecules and other small non-proteinous molecules. However, in the adaptation of PD for the selection of material specific peptides, there is a need for the fine tuning and optimization of the method. The main challenge was (and always is) that each material surface of a given has distinct surface properties. Therefore, for each material system in a specific form, PD system must be carefully optimized, each time so as to avoid non-specific binders. 

Elution of the bound phage particles from the material surfaces can be problematic, as sometimes the phage clones bound very strongly due to defects or surface chemistry. Thus not only chemical approaches, but also some physical approaches might be necessary to remove strong phage clones very efficiently [128]. Following the biopanning process, the selected peptides further need to be characterized not only for their binding affinity toward the target material but also for their selectivity. To date, there are a limited number of studies that have investigated the mode of interaction between PD selected peptides and target material systems. This challenge deserves a deeper understanding of molecular interactions of PD selected peptides with materials surfaces. Another point that needs to be addressed is that some studies do not employ a set of negative control groups to demonstrate use and applications of the particles under investigation. This is important to the specificity as well as the affinity of the PD selected peptide within the material system given the targeted application as their most remarkable feature.

PD display has made use of Nature’s way of material evolution to create new generation of materials with new functionality. To date remarkable progress has been made in the discovery and utilization of material specific peptides, which has brought new challenges and opportunities. Despite some problems in the selection and application of such PD selected material binding peptides, they promise a wide range of unusual applications in nanotechnology. 

## References

[1] Smith G.P. (1985). Filamentous Fusion Phage - Novel Expression Vectors That Display Cloned Antigens on the Virion Surface. Science.

[2] Pande J., Szewczyk M.M., Grover A.K. (2010). Phage display: Concept, innovations, applications and future. Biotechnol. Adv..

[3] Rader C., Barbas C.F. (1997). Phage display of combinatorial antibody libraries. Curr. Opin. Biotechnol..

[4] Dunn I.S. (1996). Phage display of proteins. Curr. Opin. Biotechnol..

[5] Sarikaya M., Tamerler C., Jen A.K.Y., Schulten K., Baneyx F. (2003). Molecular biomimetics: nanotechnology through biology. Nat. Mater..

[6] Slocik J.M., Naik R.R. (2010). Probing peptide-nanomaterial interactions. Chem. Soc. Rev..

[7] Sethi M., Pacardo D.B., Knecht M.R. (2010). Biological Surface Effects of Metallic Nanomaterials for Applications in Assembly and Catalysis. Langmuir.

[8] Bratkovic T. (2010). Progress in phage display: Evolution of the technique and its applications. Cell. Mol. Life Sci..

[9] Huovinen T., Sanmark H., Yla-Pelto J., Vehniainen M., Lamminmaki U. (2010). Oligovalent Fab Display on M13 Phage Improved by Directed Evolution. Mol. Biotechnol..

[10] Alexander P.A., Rozak D.A., Orban J., Bryan P.N. (2005). Directed evolution of highly homologous proteins with different folds by phage display: Implications for the protein folding code. Biochemistry.

[11] Hu Y. (2003). Biomimetic strategy for antifouling materials developed from mussel adhesive protein mimetic polymers. Mrs Bull..

[12] Holzhuter G., Lakshminarayanan K., Gerber T. (2005). Silica structure in the spicules of the sponge Suberites domuncula. Anal. Bioanal. Chem..

[13] Yang H.T.Y., Lin C.H., Bridges D., Randall C.J., Hansma P.K. (2010). Bio-inspired passive actuator simulating an abalone shell mechanism for structural control. Smart Mater. Struct..

[14] Mann S. (1997). The biomimetics of enamel: A paradigm for organized biomaterials synthesis. Ciba Foundation Symp..

[15] Wang X.H., Schroder H.C., Muller W.E.G. (2009). Giant Siliceous Spicules from the Deep-Sea Glass Sponge Monorhaphis Chuni. Int. Rev. Cell. Mol. Biol..

[16] Marin F., Luquet G., Marie B., Medakovic D. (2008). Molluscan shell proteins: Primary structure, origin, and evolution. Curr. Top. Dev. Biol..

[17] Busch S. (2004). Regeneration of human tooth enamel. Angew. Chem. Int. Ed..

[18] Baldassarri M., Margolis H.C., Beniash E. (2008). Compositional determinants of mechanical properties of enamel. J. Dent. Res..

[19] Fong H., Foster B.L., Sarikaya M., Somerman M.J. (2009). Structure and mechanical properties of Ank/Ank mutant mouse dental tissues--an animal model for studying periodontal regeneration. Arch. Oral Biol..

[20] Metzler R.A., Evans J.S., Killian C.E., Zhou D., Churchill T.H., Appathurai N.P., Coppersmith S.N., Gilbert P.U. (2010). Nacre protein fragment templates lamellar aragonite growth. J. Am. Chem. Soc..

[21] Lin A.Y., Chen P.Y., Meyers M.A. (2008). The growth of nacre in the abalone shell. Acta Biomater..

[22] Salih E., Wang J.X., Mah J., Fluckiger R. (2002). Natural variation in the extent of phosphorylation of bone phosphoproteins as a function of *in vivo* new bone formation induced by demineralized bone matrix in soft tissue and bony environments. Biochem. J..

[23] Alves N.M., Leonor I.B., Azevedo H.S., Reis R.L., Mano J.F. (2010). Designing biomaterials based on biomineralization of bone. J. Mater. Chem..

[24] Mahamid J., Aichmayer B., Shimoni E., Ziblat R., Li C.H., Siegel S., Paris O., Fratzl P., Weiner S., Addadi L. (2010). Mapping amorphous calcium phosphate transformation into crystalline mineral from the cell to the bone in zebrafish fin rays. Proc. Natl. Acad. Sci. USA.

[25] George A., Ravindran S. (2010). Protein templates in hard tissue engineering. Nano Today.

[26] Lakshminarayanan R., Vivekanandan S., Samy R.P., Banerjee Y., Chi-Jin E.O., Teo K.W., Jois S.D.S., Kini R.M., Valiyaveettil S. (2008). Structure, self-assembly, and dual role of a beta-defensin-like peptide from the chinese soft-shelled turtle eggshell matrix. J. Am. Chem. Soc..

[27] He G., Dahl T., Veis A., George A. (2003). Nucleation of apatite crystals in vitro by self-assembled dentin matrix protein, 1. Nat. Mater..

[28] Shen X.Y., Belcher A.M., Hansma P.K., Stucky G.D., Morse D.E. (1997). Molecular cloning and characterization of lustrin A, a matrix protein from shell and pearl nacre of Haliotis rufescens. J. Biol. Chem..

[29] Matsunaga T., Suzuki T., Tanaka M., Arakaki A. (2007). Molecular analysis of magnetotactic bacteria and development of functional bacterial magnetic particles for nano-biotechnology. Trends Biotechnol..

[30] Gotliv B.A., Kessler N., Sumerel J.L., Morse D.E., Tuross N., Addadi L., Weiner S. (2005). Asprich: A novel aspartic acid-rich protein family from the prismatic shell matrix of the bivalve Atrina rigida. Chembiochem.

[31] Poulsen N., Sumper M., Kroger N. (2003). Biosilica formation in diatoms: characterization of native silaffin-2 and its role in silica morphogenesis. Proc. Natl. Acad. Sci. USA.

[32] Brown S. (1997). Metal-recognition by repeating polypeptides. Nat. Biotechnol..

[33] Brown S. (1992). Engineered iron oxide-adhesion mutants of the Escherichia coli phage lambda receptor. Proc. Natl. Acad. Sci. USA.

[34] Sarikaya M., Tamerler C., Schwartz D.T., Baneyx F.O. (2004). Materials assembly and formation using engineered polypeptides. Annu. Rev. Mater. Res..

[35] Smith G.P., Petrenko V.A. (1997). Phage display. Chem. Rev..

[36] Efimov V.P., Nepluev I.V., Mesyanzhinov V.V. (1995). Bacteriophage-T4 as a Surface Display Vector. Virus Genes.

[37] Sternberg N., Hoess R.H. (1995). Display of Peptides and Proteins on the Surface of Bacteriophage-Lambda. Proc. Natl. Acad. Sci. USA.

[38] Kriplani U., Kay B.K. (2005). Selecting peptides for use in nanoscale materials using phagedisplayed combinatorial peptide libraries. Curr. Opin. Biotechnol..

[39] Tamerler C., Sarikaya M. (2009). Molecular biomimetics: nanotechnology and bionanotechnology using genetically engineered peptides. Phil. Trans. Roy. Soc. A-Math. Phys. Eng. Sci..

[40] Whaley S.R., English D.S., Hu E.L., Barbara P.F., Belcher A.M. (2000). Selection of peptides with semiconductor binding specificity for directed nanocrystal assembly. Nature.

[41] Sano K., Shiba K. (2003). A hexapeptide motif that electrostatically binds to the surface of titanium. J. Am. Chem. Soc..

[42] Park T.J., Lee S.Y., Lee S.J., Park J.P., Yang K.S., Lee K.B., Ko S., Park J.B., Kim T., Kim S.K., Shin Y.B., Chung B.H., Ku S.J., Kim D.H., Choi I.S. (2006). Protein nanopatterns and biosensors using gold binding polypeptide as a fusion partner. Anal. Chem..

[43] Cesareni G. (1992). Peptide Display on Filamentous Phage Capsids - a New Powerful Tool to Study Protein Ligand Interaction. FEBS Lett..

[44] Hayashi T., Sano K., Shiba K., Kumashiro Y., Iwahori K., Yamashita I., Hara M. (2006). Mechanism underlying specificity of proteins targeting inorganic materials. Nano Lett..

[45] Estephan E., Larroque C., Bec N., Martineau P., Cuisinier F.J., Cloitre T., Gergely C. (2009). Selection and mass spectrometry characterization of peptides targeting semiconductor surfaces. Biotechnol. Bioeng..

[46] Seker U.O., Wilson B., Sahin D., Tamerler C., Sarikaya M. (2009). Quantitative affinity of genetically engineered repeating polypeptides to inorganic surfaces. Biomacromolecules.

[47] Morita Y., Ohsugi T., Iwasa Y., Tamiya E. (2004). A screening of phage displayed peptides for the recognition of fullerene (C60). J. Mol. Catal. B-Enzym..

[48] Armitage D.A., Parker T.L., Grant D.M. (2003). Biocompatibility and hemocompatibility of surface-modified NiTi alloys. J. Biomed. Mater. Res. Part A.

[49] Colic M., Rudolf R., Stamenkovic D., Anzel I., Vucevic D., Jenko M., Lazic V., Lojen G. (2010). Relationship between microstructure, cytotoxicity and corrosion properties of a Cu-Al-Ni shape memory alloy. Acta Biomater..

[50] Nochomovitz R., Amit M., Matmor M., Ashkenasy N. (2010). Bioassisted multi-nanoparticle patterning using single-layer peptide templates. Nanotechnology.

[51] Brown S., Sarikaya M., Johnson E. (2000). A genetic analysis of crystal growth. J. Mol. Biol..

[52] Park T.J., Zheng S., Kang Y.J., Lee S.Y. (2009). Development of a whole-cell biosensor by cell surface display of a gold-binding polypeptide on the gold surface. FEMS Microbiol. Lett..

[53] Huang Y., Chiang C.Y., Lee S.K., Gao Y., Hu E.L., De Yoreo J., Belcher A.M. (2005). Programmable assembly of nanoarchitectures using genetically engineered viruses. Nano Lett..

[54] Slocik J.M., Stone M.O., Naik R.R. (2005). Synthesis of gold nanoparticles using multifunctional peptides. Small.

[55] Naik R.R., Stringer S.J., Agarwal G., Jones S.E., Stone M.O. (2002). Biomimetic synthesis and patterning of silver nanoparticles. Nat. Mater..

[56] Kim J., Rheem Y., Yoo B., Chong Y., Bozhilov K.N., Kim D., Sadowsky M.J., Hur H.G., Myung N.V. (2010). Peptide-mediated shape- and size-tunable synthesis of gold nanostructures. Acta Biomater..

[57] Lee E., Kim D.H., Woo Y., Hur H.G., Lim Y. (2008). Solution structure of peptide AG4 used to form silver nanoparticles. Biochem. Biophys. Res. Commun..

[58] Naik R.R., Jones S.E., Murray C.J., McAuliffe J.C., Vaia R.A., Stone M.O. (2004). Peptide templates for nanoparticle synthesis derived from polymerase chain reaction-driven phage display. Adv. Funct. Mater..

[59] Sengupta A., Thai C.K., Sastry M.S.R., Matthaei J.F., Schwartz D.T., Davis E.J., Baneyx F. (2008). A genetic approach for controlling the binding and orientation of proteins on nanoparticles. Langmuir.

[60] Sarikaya M., Tamerler C., Jen A.K., Schulten K., Baneyx F. (2003). Molecular biomimetics: nanotechnology through biology. Nat. Mater..

[61] Kantarci N., Tamerler C., Sarikaya M., Haliloglu T., Doruker P. (2005). Molecular dynamics simulations on constraint metal binding peptides. Polymer.

[62] Oren E.E., Tamerler C., Sarikaya M. (2005). Metal recognition of septapeptides via polypod molecular architecture. Nano Lett..

[63] Seker U.O.S., Wilson B., Dincer S., Kim I.W., Oren E.E., Evans J.S., Tamerler C., Sarikaya M. (2007). Bn, Adsorption behavior of linear and cyclic genetically engineered platinum binding peptides. Langmuir.

[64] Seker U.O.S., Wilson B., Sahin D., Tamerler C., Sarikaya M. (2009). Quantitative Affinity of Genetically Engineered Repeating Polypeptides to Inorganic Surfaces. Biomacromolecules.

[65] Dincer S., Tamerler C., Sarikaya M., Piskin E. (2008). Photoresponsive peptide-azobenzene conjugates that specifically interact with platinum surfaces. Surf. Sci..

[66] Khatayevich D., Gungormus M., Yazici H., So C., Cetinel S., Ma H., Jen A., Tamerler C., Sarikaya M. (2010). Biofunctionalization of materials for implants using engineered peptides. Acta Biomater..

[67] Li Y., Whyburn G.P., Huang Y. (2009). Specific peptide regulated synthesis of ultrasmall platinum nanocrystals. J. Am. Chem. Soc..

[68] Sasaki K., Naohara H., Cai Y., Choi Y.M., Liu P., Vukmirovic M.B., Wang J.X., Adzic R.R. (2010). Core-Protected Platinum Monolayer Shell High-Stability Electrocatalysts for Fuel-Cell Cathodes. Angew. Chem.-Int. Ed..

[69] Pacardo D.B., Sethi M., Jones S.E., Naik R.R., Knecht M.R. (2009). Biomimetic synthesis of Pd nanocatalysts for the Stille coupling reaction. ACS Nano.

[70] Nian R., Kim D.S., Thuong N., Tan L.H., Kim C.W., Yoo I.K., Choe W.S. (2010). Chromatographic biopanning for the selection of peptides with high specificity to Pb2+ from phage displayed peptide library. J. Chromatogr. A.

[71] Zuo R.J., Ornek D., Wood T.K. (2005). Aluminum- and mild steel-binding peptides from phage display. Appl. Microbiol. Biotechnol..

[72] Liu Y., Mao J., Zhou B., Wei W., Gong S.Q. (2010). Peptide aptamers against titanium-based implants identified through phage display. J. Mater. Sci. Mater. M.

[73] Meyers S.R., Hamilton P.T., Walsh E.B., Kenan D.J., Grinstaff M.W. (2007). Endothelialization of titanium surfaces. Advan. Mater..

[74] Naik R.R., Brott L.L., Clarson S.J., Stone M.O. (2002). Silica-precipitating peptides isolated from a combinatorial phage display peptide library. J. Nanosci. Nanotechnol..

[75] Tamerler C., Kacar T., Sahin D., Fong H., Sarikaya M. (2007). Genetically engineered polypeptides for inorganics: A utility in biological materials science and engineering. Mater. Sci. Eng. C-Biomim. Supramol. Syst..

[76] Eteshola E., Brillson L.J., Lee S.C. (2005). Selection and characteristics of peptides that bind thermally grown silicon dioxide films. Biomol. Eng..

[77] Chen H.B., Su X.D., Neoh K.G., Choe W.S. (2006). QCM-D analysis of binding mechanism of phage particles displaying a constrained heptapeptide with specific affinity to SiO2 and TiO2. Anal. Chem..

[78] Chen H.B., Su X.D., Neoh K.G., Choe W.S. (2008). Probing the interaction between peptides and metal oxides using point mutants of a TiO2-binding peptide. Langmuir.

[79] Sano K.I., Sasaki H., Shiba K. (2005). Specificity and biomineralization activities of Ti-binding peptide-1 (TBP-1). Langmuir.

[80] Bonne M., Pronier S., Batonneau Y., Can F., Courtois X., Royer S., Marecot P., Duprez D. (2010). Surface properties and thermal stability of SiO2-crystalline TiO2 nano-composites. J. Mater. Chem..

[81] Sano K., Ajima K., Iwahori K., Yudasaka M., Iijima S., Yamashita I., Shiba K. (2005). Endowing a ferritin-like cage protein with high affinity and selectivity for certain inorganic materials. Small.

[82] Sano K., Sasaki H., Shiba K. (2006). Utilization of the pleiotropy of a peptidic aptamer to fabricate heterogeneous nanodot-containing multilayer nanostructures. J. Am. Chem. Soc..

[83] Sano K., Yoshii S., Yamashita I., Shiba K. (2007). In aqua structuralization of a three-dimensional configuration using biomolecules. Nano Lett..

[84] Dickerson M.B., Jones S.E., Cai Y., Ahmad G., Naik R.R., Kroger N., Sandhage K.H. (2008). Identification and design of peptides for the rapid, high-yield formation of nanoparticulate TiO_2_ from aqueous solutions at room temperature. Chem. Mater..

[85] Oren E.E., Tamerler C., Sahin D., Hnilova M., Seker U.O., Sarikaya M., Samudrala R. (2007). A novel knowledge-based approach to design inorganic-binding peptides. Bioinformatics.

[86] Notman R., Oren E.E., Tamerler C., Sarikaya M., Samudrala R., Walsh T.R. (2010). Solution Study of Engineered Quartz Binding Peptides Using Replica Exchange Molecular Dynamics. Biomacromolecules.

[87] Fang Y., Poulsen N., Dickerson M.B., Cai Y., Jones S.E., Naik R.R., Kroger N., Sandhage K.H. (2008). Identification of peptides capable of inducing the formation of titania but not silica via a subtractive bacteriophage display approach. J. Mater. Chem..

[88] Ginley D.S., Bright C. (2000). Transparent conducting oxides. Mrs Bull..

[89] Umetsu M., Mizuta M., Tsumoto K., Ohara S., Takami S., Watanabe H., Kumagai I., Adschiri T. (2005). Bioassisted room-temperature immobilization and mineralization of zinc oxide - The structural ordering of ZnO nanoparticles into a flower-type morphology. Advan. Mater..

[90] Tomczak M.M., Gupta M.K., Drummy L.F., Rozenzhak S.M., Nalk R.R. (2009). Morphological control and assembly of zinc oxide using a biotemplate. Acta Biomater..

[91] He R.L., Tsuzuki T. (2010). Low-Temperature Solvothermal Synthesis of ZnO Quantum Dots. J. Am. Ceram. Soc..

[92] Vreuls C., Zocchi G., Genin A., Archambeau C., Martial J., Van de Weerdt C. (2010). Inorganic-binding peptides as tools for surface quality control. J. Inorg. Biochem..

[93] Nam Y.S., Magyar A.P., Lee D., Kim J.W., Yun D.S., Park H., Pollom T.S., Weitz D.A., Belcher A.M. (2010). Biologically templated photocatalytic nanostructures for sustained light-driven water oxidation. Nat. Nanotechnol..

[94] Dickerson M.B., Naik R.R., Stone M.O., Cai Y., Sandhage K.H. (2004). Identification of peptides that promote the rapid precipitation of germania nanoparticle networks via use of a peptide display library. Chem. Commun..

[95] Lower B.H., Lins R.D., Oestreicher Z., Straatsma T.P., Hochella M.F., Shi L.A., Lower S.K. (2008). *In vitro* evolution of a peptide with a hematite binding motif that may constitute a natural metal-oxide binding archetype. Environ. Sci. Technol..

[96] Reiss B.D., Mao C.B., Solis D.J., Ryan K.S., Thomson T., Belcher A.M. (2004). Biological routes to metal alloy ferromagnetic nanostructures. Nano Lett..

[97] Lee S.K., Yun D.S., Belcher A.M. (2006). Cobalt ion mediated self-assembly of genetically engineered bacteriophage for biomimetic Co-Pt hybrid material. Biomacromolecules.

[98] Ahmad G., Dickerson M.B., Cai Y., Jones S.E., Ernst E.M., Vernon J.P., Haluska M.S., Fang Y., Wang J., Subrarnanyarn G., Naik R.R., Sandhage K.H. (2008). Rapid bioenabled formation of ferroelectric BaTiO3 at room temperature from an aqueous salt solution at near neutral pH. J. Am. Chem. Soc..

[99] Reiss B.D., Bai G.R., Auciello O., Ocola L.E., Firestone M.A. (2006). Identification of peptides for the surface functionalization of perovskite ferroelectrics. Appl. Phys. Lett..

[100] Nizamoglu S., Demir H.V. (2007). Nanocrystal-based hybrid white light generation with tunable colour parameters. J. Opt. A-Pure Appl. Opt..

[101] Medintz I.L., Konnert J.H., Clapp A.R., Stanish I., Twigg M.E., Mattoussi H., Mauro J.M., Deschamps J.R. (2004). A fluorescence resonance energy transfer-derived structure of a quantum dot-protein bioconjugate nanoassembly. Proc. Natl. Acad. Sci. USA.

[102] Estephan E., Larroque C., Cuisinier F.J.G., Balint Z., Gergely C. (2008). Tailoring GaN semiconductor surfaces with biomolecules. J. Phys. Chem. B.

[103] Flynn C.E., Mao C.B., Hayhurst A., Williams J.L., Georgiou G., Iverson B., Belcher A.M. (2003). Synthesis and organization of nanoscale II-VI semiconductor materials using evolved peptide specificity and viral capsid assembly. J. Mater. Chem..

[104] Flynn C.E., Lee S.W., Peelle B.R., Belcher A.M. (2003). Viruses as vehicles for growth, organization and assembly of materials. Acta Mater..

[105] Estephan E., Saab M.B., Larroque C., Martin M., Olsson F., Lourdudoss S., Gergely C. (2009). Peptides for functionalization of InP semiconductors. J. Colloid Interface Sci..

[106] Goede K., Busch P., Grundmann M. (2004). Binding specificity of a peptide on semiconductor surfaces. Nano Lett..

[107] Fincham A.G., Simmer J.P. (1997). Amelogenin proteins of developing dental enamel. Ciba Foundation Symp..

[108] Gungormus M., Fong H., Kim I.W., Evans J.S., Tamerler C., Sarikaya M. (2008). Regulation of in vitro calcium phosphate mineralization by combinatorially selected hydroxyapatite-binding peptides. Biomacromolecules.

[109] Roy M.D., Stanley S.K., Amis E.J., Becker M.L. (2008). Identification of a highly specific hydroxyapatite-binding peptide using phage display. Advan. Mater..

[110] Gaskin D.J.H., Starck K., Vulfson E.N. (2000). Identification of inorganic crystal-specific sequences using phage display combinatorial library of short peptides: A feasibility study. Biotechnol. Lett..

[111] Li C.M., Botsaris G.D., Kaplan D.L. (2002). Selective in vitro effect of peptides on calcium carbonate crystallization. Cryst. Growth Des..

[112] Cao Q., Kim H.S., Pimparkar N., Kulkarni J.P., Wang C.J., Shim M., Roy K., Alam M.A., Rogers J.A. (2008). Medium-scale carbon nanotube thin-film integrated circuits on flexible plastic substrates. Nature.

[113] Shannon M.A., Bohn P.W., Elimelech M., Georgiadis J.G., Marinas B.J., Mayes A.M. (2008). Science and technology for water purification in the coming decades. Nature.

[114] Chung K., Lee C.H., Yi G.C. (2010). Transferable GaN Layers Grown on ZnO-Coated Graphene Layers for Optoelectronic Devices. Science.

[115] Miller J.R., Outlaw R.A., Holloway B.C. (2010). Graphene Double-Layer Capacitor with ac Line-Filtering Performance. Science.

[116] Wang S., Humphreys E.S., Chung S.Y., Delduco D.F., Lustig S.R., Wang H., Parker K.N., Rizzo N.W., Subramoney S., Chiang Y.M., Jagota A. (2003). Peptides with selective affinity for carbon nanotubes. Nat. Mater..

[117] Walsh T.R., Tomasio S.M. (2010). Investigation of the influence of surface defects on peptide adsorption onto carbon nanotubes. Mol. Biosyst..

[118] Kulp J.L., Shiba K., Evans J.S. (2005). Probing the conformational features of a phage display polypeptide sequence directed against single-walled carbon nanohorn surfaces. Langmuir.

[119] Sanghvi A.B., Miller K.P., Belcher A.M., Schmidt C.E. (2005). Biomaterials functionalization using a novel peptide that selectively binds to a conducting polymer. Nat. Mater..

[120] Serizawa T., Sawada T., Matsuno H., Matsubara T., Sato T. (2005). A peptide motif recognizing a polymer stereoregularity. J. Am. Chem. Soc..

[121] Serizawa T., Sawada T., Matsuno H. (2007). Highly specific affinities of short peptides against synthetic polymers. Langmuir.

[122] Matsuno H., Sekine J., Yajima H., Serizawa T. (2008). Biological selection of peptides for poly(L-lactide) substrates. Langmuir.

[123] Serizawa T., Techawanitchai P., Matsuno H. (2007). Isolation of peptides that can recognize syndiotactic polystyrene. Chembiochem.

[124] Serizawa T., Sawada T., Kitayama T. (2007). Peptide motifs that recognize differences in polymer-film surfaces. Angew. Chem.-Int. Ed..

[125] Serizawa T., Iida K., Matsuno H., Kurita K. (2007). Cellulose-binding heptapeptides identified by phage display methods. Chem. Lett..

[126] Nam K.T., Lee Y.J., Krauland E.M., Kottmann S.T., Belcher A.M. (2008). Peptide-mediated reduction of silver ions on engineered biological scaffolds. ACS Nano.

[127] Togashi T., Yokoo N., Umetsu M., Ohara S., Naka T., Takami S., Abe H., Kumagai I., Adschiri T. (2010). Material-binding peptide application-ZnO crystal structure control by means of a ZnO-binding peptide. J. Biosci. Bioeng..

[128] Donatan S., Yazici H., Bermek H., Sarikaya M., Tamerler C., Urgen M. (2009). Physical elution in phage display selection of inorganic-binding peptides. Mater. Sci. Eng. C-Biomim. Supramol. Syst..

[129] Hayashi T., Sano K.I., Shiba K., Iwahori K., Yamashita I., Hara M. (2009). Critical Amino Acid Residues for the Specific Binding of the Ti-Recognizing Recombinant Ferritin with Oxide Surfaces of Titanium and Silicon. Langmuir.

[130] Nam K.T., Kim D.W., Yoo P.J., Chiang C.Y., Meethong N., Hammond P.T., Chiang Y.M., Belcher A.M. (2006). Virus-enabled synthesis and assembly of nanowires for lithium ion battery electrodes. Science.

[131] Naik R.R., Stringer S.J., Agarwal G., Jones S.E., Stone M.O. (2002). Biomimetic synthesis and patterning of silver nanoparticles. Nat. Mater..

[132] Eteshola E., Brillson L.J., Lee S.C. (2005). Selection and characteristics of peptides that bind thermally grown silicon dioxide films. Biomol. Eng..

[133] Chen H.B., Su X.D., Neoh K.G., Choe W.S. (2009). Engineering Lacl for Self Assembly of Inorganic Nanoparticles on DNA Scaffold through the Understanding of Lacl Binding to Solid Surfaces. Adv. Funct. Mater..

[134] Chiu C.Y., Li Y.J., Huang Y. (2010). Size-controlled synthesis of Pd nanocrystals using a specific multifunctional peptide. Nanoscale.

[135] Date T., Tanaka K., Nagamura T., Serizawa T. (2008). Directional affinity of short peptides for synthetic polymers. Chem. Mater..

[136] Kacar T., Ray J., Gungormus M., Oren E.E., Tamerler C., Sarikaya M. (2009). Quartz Binding Peptides as Molecular Linkers towards Fabricating Multifunctional Micropatterned Substrates. Advan. Mater..

[137] Zin M.T., Munro A.M., Gungormus M., Wong N.Y., Ma H., Tamerler C., Ginger D.S., Sarikaya M., Jen A.K.Y. (2007). Peptide-mediated surface-immobilized quantum dot hybrid nanoassemblies with controlled photoluminescence. J. Mater. Chem..

[138] Cui Y., Kim S.N., Jones S.E., Wissler L.L., Naik R.R., McAlpine M.C. (2010). Chemical Functionalization of Graphene Enabled by Phage Displayed Peptides. Nano Lett..

[139] Kuang Z., Kim S.N., Crookes-Goodson W.J., Farmer B.L., Naik R.R. (2010). Biomimetic chemosensor: designing peptide recognition elements for surface functionalization of carbon nanotube field effect transistors. ACS Nano.

[140] Mao C., Solis D.J., Reiss B.D., Kottmann S.T., Sweeney R.Y., Hayhurst A., Georgiou G., Iverson B., Belcher A.M. (2004). Virus-based toolkit for the directed synthesis of magnetic and semiconducting nanowires. Science.

[141] Lee Y.J., Yi H., Kim W.J., Kang K., Yun D.S., Strano M.S., Ceder G., Belcher A.M. (2009). Fabricating genetically engineered high-power lithium-ion batteries using multiple virus genes. Science.

